# Muscle-driven forward dynamic simulations for the study of normal and pathological gait

**DOI:** 10.1186/1743-0003-3-5

**Published:** 2006-03-06

**Authors:** Stephen J Piazza

**Affiliations:** 1 Departments of Kinesiology, Mechanical Engineering, and Orthopaedics and Rehabilitation, University Park, PA and Hershey, PA, USA

## Abstract

There has been much recent interest in the use of muscle-actuated forward dynamic simulations to describe human locomotion. These models simulate movement through the integration of dynamic equations of motion and usually are driven by excitation inputs to muscles. Because motion is effected by individual muscle actuators, these simulations offer potential insights into the roles played by muscles in producing walking motions. Better knowledge of the actions of muscles should lead to clarification of the etiology of movement disorders and more effective treatments. This article reviews the use of such simulations to characterize musculoskeletal function and describe the actions of muscles during normal and pathological locomotion. The review concludes by identifying ways in which models must be improved if their potential for clinical utility is to be realized.

## Introduction

Gait disorders are often attributed either to muscles interfering with locomotor function or to muscles being prevented from performing their proper actions. Many options are available for addressing problems with individual muscles, including tendon transfers, tendon lengthenings, osteotomies, and localized treatment with pharmacological agents, but it is not always easy to identify candidate muscles for treatment and the effects of these treatments on gait are often unpredictable.

Identification of the root causes of gait abnormalities is difficult because the locomotor apparatus is so complex. It is usually the case that multiple joints and multiple muscles are involved and the clinician is often required to separate the primary gait abnormality from secondary compensatory mechanisms adopted by the patient. Clinical gait analysis provides a great deal of information that can aid in the selection of an appropriate treatment, but the function or dysfunction of individual muscles is often not clearly determined from joint kinematic and kinetic data. For example, a patient with insufficient knee flexion during the swing phase may have knee flexion limited by several potential mechanisms, including: overactive quadriceps that restrain knee flexion; weak hip flexors that fail to advance the thigh; an inadequate push-off that does not start the knee flexing fast enough in terminal stance; or some combination of all of these factors. While traditional inverse dynamic analysis can identify excessive or abnormally small joint moments, such an analysis cannot predict the effects of altered muscle action on movement, or decompose a movement into its component determinants. A more complete assessment of the patient's gait problems would require consideration of the roles played by muscles, gravity, and intersegment reaction forces, all occurring in three dimensions.

The purpose of the present paper is to review the application of one form of computer simulation, *forward dynamic musculoskeletal simulation*, to the study of normal and pathological walking. Much excellent work has been published that describes the development of techniques that have made simulations of movement faster or more accurate, but this review is focused on clinical applications rather than modeling methods. The reader interested in a more general treatment of technical advances in musculoskeletal modeling and simulation is referred to Yamaguchi's textbook [1] and to previous reviews by Zajac et al. [2-4], Neptune [5], Hatze [6], and Pandy [7].

## Forward dynamic musculoskeletal simulation

In a forward dynamic simulation, differential equations of motion are numerically integrated forward in time subject to gravity, inertial and velocity-dependent effects, and muscle forces. It is a 'forward' simulation in the sense that forces produce motions and is distinct from inverse dynamic analyses in which internal (muscular) moments are computed from measured motions and external forces. One advantage of solving for motion through numerical integration of equations of motion rather than applying conditions of equilibrium in a static or quasi-static formulation is that there is no theoretical limitation on the number of degrees of freedom or the number of unknown forces that must be determined. If state equations can be written that describe the multibody dynamics of the body segments and joints as well as the computation of forces applied to those segments, then those equations can be used to predict positions and velocities going forward from some initial state.

A dizzying array of technical choices is made when forward dynamic simulations like the ones described in this review are created. A partial list includes the following: the number of degrees of freedom in the model; the body segment inertial parameters; kinematic behavior of the joints; the bony geometry and muscle attachment locations; the mathematical model of muscle force generation; the muscle force generating properties; modeling of ligamentous restraints to joint motion; modeling of contact between the feet and the ground; modeling of contact within the joints; the method used to integrate the equations of motion. Also important is the scheme used to arrive at the set of muscle excitation inputs that drive the motion. These inputs may be derived directly from measured muscle activity, or indirectly using an optimization algorithm that minimizes some objective function such as the aggregate deviation from a given motion. Each of these choices has the potential to affect the performance of the simulation and thus may also affect the validity of clinical applications of such models.

## Model-based determination of internal forces

Knowledge of the forces carried by ligaments, tendons, and joints under normal conditions are of clinical interest but such measurements cannot be made readily without substantially invasive procedures. Computer simulation permits full monitoring of quantities such as joint contact loads and soft tissue forces. In this way, a model of the musculoskeletal system can be 'instrumented' in ways that would be impossible with a living human subject. An unlimited number of soft tissue tensions and joint contact forces may be monitored during a simulation without the slightest disturbance to the simulation output.

One example of a soft-tissue tension that is both of high clinical relevance and difficult to monitor *in vivo *is the force carried by the anterior cruciate ligament (ACL). In two recent studies, Shelburne et al. [8,9] investigated ACL loading and the mechanics of the ACL-deficient knee during gait. Two models were employed for this purpose. A three-dimensional dynamic simulation of the whole body walking was performed with a constrained, single-degree-of-freedom knee to determine joint kinematics, muscle forces, and ground reaction forces; these outputs were then used in an unconstrained static knee model to compute both the loads carried by ligaments and the translations within the knee at every timestep during the gait cycle. The authors found that the ACL carried loads throughout the stance phase and that these loads peaked early in stance. The medial collateral ligament was found to be the structure that compensated most when the ACL was removed, although the overall shear loading of the knee was reduced by changes in the anterior tibial translation.

Knee loading has also been investigated by authors using models that incorporated articular contact modeling into dynamic simulations. Contact formulations have been employed that assume the contacting bodies are rigid [10,11], employ a deformable elastic foundation on a rigid substrate [12-14], or incorporate finite-element models of contact [12]. Halloran et al. [12] reported similar results both for models of total knee replacement motions incorporating finite-element modeling of contact and for less computationally expensive elastic foundation models. Fregly et al. [14] demonstrated that dynamic simulations incorporating elastic foundation models can predict in vivo wear patterns in total knee replacement components and have the potential to develop into useful tools for implant design.

Sasaki and Neptune [15-17] used a dynamic simulation to investigate the factors that influence the walk-to-run transition. Previous investigations on this topic have not revealed an apparent kinematic or kinetic factor that triggers this gait transition in humans [18,19], though Prilusky and Gregor [20] noted differences in the electromyographic activity of flexor and extensor muscles above and below the transition speed. The simulations of Sasaki and Neptune illustrated that muscle fibers do more work during running below the transition speed than during walking at the same speed [16]. It was also found that above the transition speed, more fiber work is done during walking than during running, and that greater use was made of energy storage in tendons when running above the transition speed than below. These modeling results suggest that efficient storage and expenditure of mechanical energy on the part of muscle-tendon units plays a key role in the walk-run transition. Further simulations [15] suggested that the function of the ankle plantarflexors, in particular, are affected by gait selection near the transition speed. At walking speeds that approach the transition speed, the force-length-velocity properties of the plantarflexors make them less able to generate force. When a running gait is adopted, plantarflexor forces increase due to these muscles operating in a more favorable range.

Several authors have performed dynamic simulations to investigate potentially dangerous activities that would be unethical or impractical to study through experimentation on human subjects. Simulations of the landing phase of a side-shuffle movement [21,22] and a sidestep cutting movement [23] have been performed to identify factors that may lead to injury. Wright et al. [22], for example, used a muscle-actuated simulation to investigate the passive subtalar joint moment and subtalar joint rotations that followed from landing subject to a number of irregular floor conditions. The authors used passive nonlinear joint restraint moments at the talocrural and subtalar joints to represent ligaments and bony constraints. They found that increased plantar flexion at touchdown, rather than increased subtalar supination, was associated with subsequent sprains in a side-shuffle movement. McLean et al. [23] performed a similar analysis in which a muscle-actuated model was used to evaluate changes in knee joint loads that resulted from altered muscle activations. Knee loads exceeding a given threshold were deemed sufficient to rupture the anterior cruciate ligament. Anterior drawer forces were never found to be great enough to rupture the ligament, suggesting that valgus loading is a more likely mechanism.

## Analyses of muscle function during normal walking

A large-scale dynamic simulation of walking is only possible with an appropriate set of muscle excitation patterns that keep the model moving forward and prevent it from falling down. This set of simulation inputs is usually determined through a dynamic optimization procedure. This optimization may minimize the differences between simulated and measured motions and ground reaction forces, but other choices for the performance criterion can provide useful information about walking mechanics. Noting that the energetic cost of locomotion (energy consumed per unit distance travelled) has a minimum near the preferred walking speed in humans, Anderson and Pandy [24] created a performance criterion that represented the metabolic energy consumed by all muscles per unit distance travelled in a whole-body simulation of walking. The set of muscle excitations that minimized this energy expenditure, subject to the condition that the terminal and initial conditions be equal, resulted in a highly realistic simulation of normal gait. This work was an important advance over earlier dynamic optimization efforts employing models that included only a few muscles [25,26] or that were restricted to the sagittal plane [25,27].

Like walking humans, large-scale walking simulations are prone to falling and are thus useful for studying stability. Gerritsen et al. [28] used a dynamic simulation of walking to investigate the means by which muscles aid in recovery from perturbations to gait. The authors simulated walking using four models that were identical except for the formulation of the muscle model. The model most resistant to perturbation was a muscle-actuated model whose muscles incorporated both the force-length and force-velocity properties. This model performed better than did models with muscles lacking either of these properties or a model actuated by moments rather than muscle forces. Yamaguchi and Zajac [26] also investigated requirements for stable walking using dynamic simulations in order to identify the muscle groups needed for sustained level walking. The authors reported that walking was possible with seven muscle groups per leg and a minimum level of ankle plantarflexor strength.

Walking simulations have also be used to challenge (or confirm) traditional thinking on human locomotion. The classical theory of the determinants of normal walking proposed by Saunders et al. [29] states that there are seven characteristics of gait that minimize energy consumption by attenuating oscillations of the center of mass (COM). The results of more recent experimental studies have suggested that some of the determinants are less important than others in producing movements of the COM [30], or even that minimizing COM movements has the opposite effect of increasing the metabolic energy cost [31]. Dynamic simulation permits examination of these issues at a level not possible in experiments because it affords the investigator access to many mechanical variables of interest. Pandy and Berme [32] used a dynamic simulation to investigate contributions to the ground reaction force (and thus also the COM acceleration) by individual determinants and found pelvic list to be less important to the ground reaction force than other determinants such as stance phase knee flexion. Neptune et al. [33] used a dynamic simulation to show that muscles do substantial work in raising the COM in early stance, a finding that perhaps highlights deficiencies in simpler models of walking as an inverted pendulum [34,35].

Many investigators who have created dynamic simulations of walking have used those simulations to characterize the actions of individual muscles. One way in which this is accomplished is by examining the accelerations produced by muscle forces. During the simulation, accelerations are produced by forces acting in combination: multiple muscle forces, gravity, and ground reaction forces, for example. To determine the accelerations produced by a single muscle force acting in isolation, it is necessary only to set all other forces equal to zero in the equations of motion at a given instant in time and compute the accelerations resulting from the remaining force of interest. The accelerations determined using such an induced acceleration analysis (IAA) may be rotational accelerations at joints or the linear accelerations of points such as the body's COM. Zajac et al. [3] importantly noted that the induced acceleration computation does not require a simulation; it is made instantaneously using the equations of motion. The value of the simulation is that it produces the history of model kinematics and forces necessary to make induced acceleration computations at any instant during the gait cycle. An alternate method for assessment of muscle roles is to compute the amount each muscle contributes to the power of individual body segments.

Neptune et al. [36] used IAA and segmental power analysis to differentiate between the roles of gastrocnemius and soleus during the stance phase of normal walking. Though these muscles are often grouped together functionally as plantarflexors of the ankle, important differences were discovered between the function of the biarticular gastrocnemius and the uniarticular soleus. While both muscles contribute to vertical support of the trunk, in mid-stance gastrocnemius increases the stance leg energy and restrains the forward motion of the trunk; soleus has the opposite effects. In late stance, the initiation of swing was found to be due to gastrocnemius alone. Anderson and Pandy [37] also found that the plantarflexors contributed to trunk support. By decomposing the ground reaction force (which is directly related to the acceleration of the COM) into its components due to individual muscles, it was possible to determine that the second peak in the vertical ground reaction force was caused by the plantarflexors, while the first peak was caused by knee and hip extensors. In a second study by Neptune et al. [38], similar roles were identified for the vasti and gluteals in early stance, and the plantarflexors were found to contribute most to a net forward muscular acceleration of the trunk in late stance. Riley et al. [39] reached a different conclusion regarding the role of the plantarflexors in propulsion, but their study examined the accelerations of the hip rather than the trunk COM [40].

Dynamic simulation has also been used to investigate the determinants of knee flexion during the swing phase of gait. Piazza and Delp [41] used a dynamic simulation to perform sensitivity analyses and IAA that indicated that hip flexion moment and the knee flexion velocity at toe-off contribute to knee flexion later in swing. Knee extension moment had the expected effect of reducing knee flexion, but the role of the biarticular rectus femoris was less clear. IAA revealed that rectus femoris provided a slight restraint to knee flexion in early swing. Anderson et al. [42] integrated the induced accelerations and initial velocities during the early part of swing phase to arrive at 'induced positions', the contribution of individual components to later joint rotation. The toe-off knee flexion velocity was found to be the major determinant of subsequent knee flexion in swing, with some muscles aiding in knee flexion and others having the opposite action. In a second study by Goldberg et al. [43], the authors investigated the factors influencing knee flexion velocity in late stance by altering the forces carried by each muscle and observing the resulting change in velocity. Vasti, rectus femoris, and soleus were all identified as potentially limiting of knee flexion velocity, while extra force applied by iliopsoas and gastrocnemius were found to increase knee flexion.

These studies provide helpful characterizations of normal gait that have implications for the identification of problems in pathological gait. For example, if hip flexor force is found to be an important determinant of the toe-off knee flexion velocity and of knee flexion in swing phase, then hip flexor weakness is implicated as a potential cause of stiff knee gait, in which knee flexion during swing phase is lacking [41,43]. An alternate approach would be to proceed directly to simulations of pathological gait in order to directly assess its causes.

## Simulations of pathological gait

It is possible to recreate the gaits of patients with movement disorders by forcing the simulation to track experimentally measured kinematic and kinetic data [44-49]. The result is a reproduction of the pathological gait pattern that can be examined using the same IAA and power analyses employed to study normal walking.

Although the most common surgical treatment for stiff knee gait is rectus femoris transfer to reduce knee extension moment, the results of dynamic simulations have suggested that this gait disorder is potentially caused by several factors [44-46]. Riley and Kerrigan [44] created subject-specific simulations of patients with stiff knee gait and found abnormal induced rotational accelerations at the knee that could result from abnormalities at either the hip or ankle, but results varied widely across patients. Goldberg et al. [46] also created models of individual patients' stiff knee gaits, finding that only one of 18 limbs displayed an abnormally large knee extension moment, while 15 of 18 exhibited reduced knee flexion velocity at toe-off.

Simulations of individual patients' gaits were also created by Higginson et al. [47] and Siegel et al. [48] to investigate coordination and control in subjects with gait disorders. Higginson et al. compared simulations of subjects with post-stroke hemiparesis to simulations of speed-matched controls, and found that support of the body weight was achieved by using an altered strategy that compensated for abnormal contributions from affected muscles. A similar study by Siegel et al. involved simulations of the individual gaits of patients with quadriceps weakness. IAA revealed that subjects used different strategies to produce knee extension when it could not be obtained from knee extensors directly.

Recreations of pathological gait patterns were also used by Arnold et al. [49], who analyzed the muscular contributions to knee flexion and extension accelerations in simulations of normal gait in order to study potential causes of crouch gait. The results suggested that the increased knee flexion that characterizes crouch gait may be caused by weakness in hip extensors, knee extensors, or soleus. Hamstrings spasticity is frequently cited as a cause of crouch gait and the hamstrings are often lengthened to treat this condition, but the hamstrings in the simulation were found to produce a small knee extension acceleration during mid-stance.

## Future progress in creating clinical applicable simulations

Although dynamic musculoskeletal simulation of human locomotion is usually driven by clinical questions, much more work has been done in creating simulations of normal gait than pathological gait. There are several good reasons for this: the walking patterns of healthy people are well-defined and stereotypical, making it easy to know when the simulated gait approximates a normal pattern; there are much more data upon which to base models of joints and muscles for young, healthy subjects; it is difficult to create the subject-specific models necessary to models the gaits of individual patients; there are performance criteria that seem to produce the correct excitation patterns for normal gait, but it is unclear what, if anything, is optimized in pathological walking. At present, dynamic simulations are used only as descriptive tools that provide insight into the mechanics of locomotion that is not possible with motion analysis and inverse dynamics alone. Dynamic simulations can provide information about the roles played by muscles in replications of normal and disordered gait, and can be used to estimate quantities not easily measured in experiments. In the future, however, it may be possible to use simulation as a preoperative planning tool used to predict the effects of surgery in a specific patient.

There is much promising work being done that will permit more realistic simulations of normal gait and that will hasten the development of accurate models of the gait of patients with movement disorders. More research is needed in the following areas if these goals are to be attained:

### • Better descriptions of the force-generating properties of muscles and ligaments

Musculotendon actuators in the musculoskeletal models used to carry out dynamic simulations are usually represented by Hill-type muscle models. The models are used to compute forces based on force-length and force-velocity relations that are scaled by a few muscle-specific parameters, such as optimal fiber length. Values of these parameters are drawn from a handful of studies on cadavers, each of which reports on only a few specimens. More such studies would be helpful in establishing normative values, but another potentially productive approach is to develop optimization schemes that will permit subject-specific force-generating properties to be determined from measured forces and motions [50-53]. Ligaments in musculoskeletal models are usually represented by torsional springs that resist excessive joint rotations, but more explicit representations will be required to predict the effects of orthopaedic surgeries on ligament tensions.

### • More complex models of muscle geometry and architecture

For the most part, muscles have been modelled as independent actuators that follow straight, segmented, or curved paths from origin to insertion. They may wrap around objects intended to represent bones, retinacula, or other muscles, but more work is necessary to account for the complex architecture of some muscles [54-57] and for mechanical and neurological coupling between muscles [58,59]. Fascial connections and synergistic activation of muscle groups are potentially important constraints not included in current models.

### • More complex models of joints

In nearly all of the simulations described in this review, the knee is represented as a single-degree-of-freedom joint whose translations and rotations are either held fixed or prescribed as functions of the knee flexion angle, a description at odds with the behavior of actual knees that exhibit a substantial degree of laxity even when healthy. The ankles are represented by a pair of fixed, skewed hinges, but we know that one of these joints, the talocrural, changes its orientation as the joint rotates, and the other, the subtalar, exhibits a high degree of intersubject variability in its orientation [60]. We know that mobility in the joints of the foot is important to normal locomotion, but the foot is usually modeled as a rigid block. Accurate descriptions of joint kinematics are especially important when tendons pass close to joint axes, as is the case at the ankle, because the moments produced by such muscles will be especially sensitive to joint position. Methods for identifying subject-specific joint kinematics [61] will help in this regard, as will studies that assess the effects of using generic joint models.

### • Means for validation

The results of dynamic simulations are likely to be sensitive to the degree of complexity in the formulation of the model [62], but most of the simulations described in this review are based on very similar musculoskeletal models. When different sets of model parameters are used to create simulations with similar results, this will provide some degree of validation in that the results will prove to be robust with respect to variation in those parameters. The extent of the validation of dynamic simulation results is usually limited to comparisons of the kinematic, kinetic, and electromyographic outputs to their experimentally-measured counterparts. Unfortunately, there is no means currently available for making a direct measurement of the acceleration produced by the action of a single muscle in human subjects, and direct validation of muscle induced acceleration analyses remains a challenge.

## Conclusion

The use of muscle-driven forward dynamic simulations to study human locomotion is becoming increasingly common; fifteen such studies cited in this article were published in the last eighteen months. A great deal of excellent work has been done to facilitate the creation of realistic simulations of walking and to begin describing the mechanics of walking in terms only possible through the use of simulation. Motion analysis describes the motions and external forces present during gait, and inverse dynamics gives further information about joint kinetics. Both of these tools are widely used in clinical gait analysis. The present challenge is to devise measures of gait performance based on dynamic simulation output and to make these measures applicable to the treatment of patients with movement disorders.
